# Family support, discrimination, and quality of life among ART-treated HIV-infected patients: a two-year study in China

**DOI:** 10.1186/s40249-017-0364-5

**Published:** 2017-11-21

**Authors:** Jun-Fang Xu, Zhong-Qiang Ming, Yu-Qian Zhang, Pei-Cheng Wang, Jun Jing, Feng Cheng

**Affiliations:** 1 Research Center for Public Health, School of Medicine, Tsinghua University, Beijing, China; 2 Fukangda Health International Science & Technology, Beijing, China; 3 The Paul H. Nitze School of Advanced International Studies, The Johns Hopkins University, Baltimore, USA; 4 Department of Sociology, Tsinghua University, Beijing, China; 5 Center for Global Health and Infectious Diseases of Tsinghua University, Beijing, China

**Keywords:** Quality of life, Family support, Discrimination, People infected with HIV, China

## Abstract

**Background:**

By September 2016, approximately 653,865 people in China were living with HIV/AIDS (PLWHA) and 492,725 people were receiving antiretroviral therapy (ART). PLWHA frequently experience discrimination in all domains of their personal and social lives. The World Health Organization includes discrimination in its list of social determinants of health factors that have been linked to poor physical and psychological health. This paper identifies the family support enjoyed and discrimination faced by people infected with HIV and examines the effect they have on patients’ quality of life (QOL) as they undergo ART in China.

**Methods:**

We conducted this observational cohort study of ART**-**treated patients with HIV in Guangxi Province using a questionnaire survey at baseline, 6, 12, and 24 months, starting in 2010. Descriptive analysis was used to describe the demographic characteristics (e.g., age, sex, educational level, marital status, and employment status) of participants. Generalized estimating equations (GEE) were employed to examine the relationships between family support, discrimination, and QOL.

**Results:**

In the study, 90.4% (*n* = 281) of patients received family support at baseline, here defined as the initiation of ART, 91.8% (*n* = 244) received family support 6 months into ART, 95.5% (*n* = 220) at 12 months, and 94.3% (*n* = 230) at 24 months. The proportion of patients who did not feel discriminated against by their families was 87.2% (*n* = 274) at baseline, 90.4% (*n* = 229) 6 months into ART, 90.0% (*n* = 210) at 12 months, and 94.5% (*n* = 219) at 24 months. Patients’ overall QOL scores were positively associated with having received family support (*OR* = 2.74, *P* = 0.040, 95% *CI*: 1.68–4.47), not feeling discriminated against by their families (*OR* = 1.3, *P* = 0.041, 95% *CI*: 1.07–1.59) or discrimination from patients themselves, including never experiencing fear of abandonment by family (*OR* = 2.05, *P* = 0.025, 95% *CI*: 1.49–2.82).

**Conclusions:**

Family support along with no or minimal discrimination was found to contribute to QOL among people infected with HIV. Their overall QOL tended to improve significantly as ART continued. This suggests that strategies meant to improve and strengthen family support, care for PLWHA, and promote HIV screening among high-risk populations should be explored by both policy makers and researchers.

**Electronic supplementary material:**

The online version of this article (10.1186/s40249-017-0364-5) contains supplementary material, which is available to authorized users.

## Multilingual abstracts

Please see Additional file 1 for translations of the abstract into the five official working languages of the United Nations.

## Background

The Chinese Center for Disease Control and Prevention estimated that the prevalence of HIV remained greater than 0.4%. As of September 2016, approximately 653,865 people in China were living with HIV/AIDS (PLWHA) and 492,725 patients were receiving antiretroviral therapy (ART) [1, 2]. About 15% of all PLWHA are young people aged 15 to 24, among whom the incidence of HIV increased from 6.9% in 2006 to 23% in 2012 (www.ncstdc.org). The average annual growth in the rate of 15 to 24 year old students infected with HIV reached 35% in 2015, and 65% of these students infected with HIV were 18–22 years old and studying at universities. This may be because young students’ minds are open but they lack sexual knowledge and have limited capacity for self-examination and self-awareness [1]. The infection rate in China’s south-western provinces and autonomous region, Yunnan, Guangxi, and Sichuan, is especially high. These three provinces are home to 12.9% of China’s total population, but they account for 45% of all PLWHA in the entire country [3]. Guangxi Zhuang Autonomous Region sees more than 5000 new cases of HIV every year, with an incidence of 0.1% [3]. In 2003, a new administration led by President Hu Jintao, Premier Wen Jiabao, and Vice Premier and Health Minister Wu Yi substantially accelerated the commitment to and implementation of evidence-based HIV policies. Under this administration, a number of initiatives were introduced, including the “Four Free and One Care” policy in December 2003, which provides free antiretroviral drugs to AIDS patients who live in rural areas and those without insurance who live in urban areas, free voluntary counselling and testing, free drugs to HIV-infected pregnant women to prevent mother-to-child transmission, HIV testing of new-born babies, free schooling for AIDS orphans, and care and economic assistance to the households of people living with HIV/AIDS. It has expanded to more than 120 sites nationwide with the aim of controlling the spread of AIDS. Special funds have been earmarked for funding highly active ART for HIV patients. In China, sexual transmission (i.e., homosexual transmission and heterosexual transmission) and the sharing needles for illegal drug use are the main causes of infection [3].

Ever since the human immunodeficiency virus (HIV) was first identified, indicators of quality of life (QOL) of the individuals carrying it and the mortality rates of AIDS patients have undergone substantial changes. The advent of combined antiretroviral therapy has contributed significantly to those changes [4–6], and highly active ART allows PLWHA to live longer. However, the diagnosis of HIV infection and subsequent antiretroviral treatment often cause both physical and emotional suffering, which diminish the patient’s QOL [7–9]. Moreover, multiple sources of stress, such as the disease itself, financial burdens, stigma, discrimination and pressure from worrying about family reactions and needs, have negative effects on the QOL of PLWHA. Preventive behaviours such as condom use, HIV test-seeking behaviour, and care-seeking behaviour upon diagnosis can also be affected by the patient’s negative feelings [10–12].

Clinical and empirical findings suggest that family can be a significant source of stress for PLWHA, second only to that of the disease itself [13, 14]. However, strong support and little stress from family may increase the QOL of PLWHA. They can also inform the design of family-cantered HIV-related interventions by increasing the participation rate and level of adherence to antiretroviral treatment and by reducing the rate of loss to follow-up rate [15]. A growing number of studies have shown that, programs for PLWHA need to engage the patients’ families in order to be successful [16, 17]. Support from family and partners can also improve adherence to therapy, which is the first important factor to be addressed in the planning of HAART services [18]. Furthermore, among HIV-infected adults whose relationships between social support and psychological adjustment were examined, greater support, including financial and spiritual support, was found to reduce psychological distress, such as symptoms of depression [19, 20].

The disclosure of serostatus by PLWHA to their partners, family, and friends is an important means of reducing the incidence of HIV infection and improving HIV treatment and care [21–23]. Over the past 15 years, notification of partners’ HIV status has been a major public health strategy in HIV prevention in China [24]. However, disclosing HIV serostatus to partners, family, and friends is still a difficult decision for many HIV-infected people in the context of high level of stigma and discrimination associated with HIV status and the risk of rejection, abandonment, violence, abuse, loss of custody of children, loss of property, and ostracism, all of which are common for PLWHA, especially women [25]. Chinese society is family-oriented, which means many individual experiences become, or are inseparable from, family matters. In this way, the whole family is often stigmatized if any one member is publicly known to be HIV positive. Others may believe that the entire family engages in unhealthy or unwholesome behaviour. More often, the family is blamed for permitting the risky behaviours that led to HIV infection. Many studies have reported similar findings regarding the impact of HIV on whole families [26–28]. The lack of necessary HIV-related knowledge and skills in communicating about sensitive topics (e.g., sexual behaviours and HIV transmission) can be also a barrier to planning an appropriate and smooth disclosure [29].

After testing positive, PLWHA will often experience HIV-related stigma and discrimination from healthy family members because it is understood that risky behaviours (such as drug use and commercial sex) lead to disease and because they resent the patient for the damage done to the reputation of the entire family. However, family support is one of the most important factors affecting how patients adapt to illness [30]. The presence of support may improve psychological and emotional well-being and reduce stress [31]. Yet few studies have explored the effect of family support and discrimination on PLWHA’s QOL in China. This study analyses family support for and discrimination against people infected with HIV undergoing ART at four different points in time and examines the relationship between the QOL, family support, and discrimination.

## Methods

### Setting and study design

An observational cohort study of patients initiating ART was conducted in five hospitals and clinics located in Guangxi Zhuang Autonomous Region in 2010, which treat 11.3% of PLWHA in China: Pingxiang People’s Hospital ART Clinic, Guangxi CDC ART Clinic in Nanning City, the 4th Nanning Hospital ART Clinic, Guigang City People’s Hospital ART Department, and Guilin 3rd People’s Hospital. Since Guangxi began ART in 2004, good progress has been made. We selected five health facilities based on the following criteria: (1) Whether they provide ART services as stipulated in China’s National ART Guidelines; (2) whether the patients there are willing to participate in the study and adhere to its protocol; (3) whether there had been an adequate number of outpatients, here an average number of ten or more over the previous three months.

Male and female people infected with HIV aged 18 years or older who had initiated ART from the one of the five clinics mentioned above were eligible to participate in the study. People infected with HIV were invited to participate and provided an informed consent form to complete if they agreed to participate in the study. Specific criteria for participation in the cohort study were as follows: (1) They had to meet ART admission criteria as stipulated in national ART guidelines; (2) HIV-positive status and undergoing ART for the first time during the recruitment period; (3) willingness to participate and provide a signed informed consent form; (4) sufficient physical capacity to complete questionnaire surveys, as assessed by the physicians in the hospitals or clinics. China’s national ART admission criteria included the following: (1) Confirmed HIV positive status; (2) meeting the medical criteria for admission (clinical and laboratory); (3) sufficient preparation for treatment (including clinical, adherence, education, and family and peer support).

**Fig. 1:**
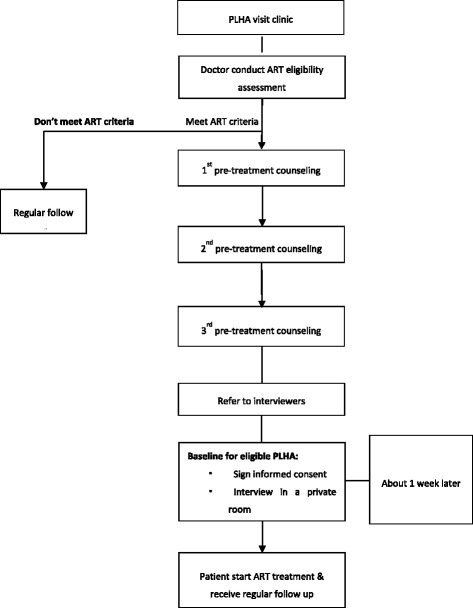
Detailed procedures by which participants were recruited

All new patients who began ART were screened during normal work hours to determine if they met the inclusion criteria and, if eligible, were invited to participate in the study. Patients who expressed an interest in participating in the study to their attending physician were referred to an interviewer, who read them the standard informed consent in the ART clinic. Patients were asked to sign the consent form if they agreed to participate. After completing the informed consent procedure, the participant was then given the detailed baseline and follow-up questionnaires in a face-to-face interview in a private setting. A total of 332 subjects were recruited for the study. To protect the patients’ privacy, all interviews were conducted in a small room in the hospital or clinic. Patients underwent regular follow-up and appointments were made with them to complete the 6-month, 12-month, and 24-month surveys. The interviewer then checked the hard copies of the questionnaires for accuracy and completeness within 1–2 days. Whenever possible, any errors in questionnaire administration were addressed by discussion with the participants by telephone. The interviewer would contact the study participant and confirm schedules for both the initial and follow-up visits on an ongoing basis and develop a spreadsheet to keep track of those appointments. Figure 1 shows the detailed procedures through which participants were recruited.

### Data collection

Structured questionnaires were developed specifically for this study. They included the following key components: demographics and socioeconomics, health-related quality of life (HR-QOL), and family support and discrimination, which included whether the participant told his or her the family he or she was positive for HIV, what support does the family provided, whether the participant felt that his or her family had discriminated against him or her, and similar questions. Quality of life data were obtained using the Chinese version of the WHOQOL-HIV BREF instrument. The WHOQOL-HIV-Brief (Chinese version) is a cross-cultural instrument that contains 31 items developed from the original 100-term questionnaire. It contains six domains, physicality, psychology, independence, social relationships, environment, and spirituality. Each item has a five-point ordinal response scale scoring from 1 to 5. Higher scores indicate better quality of life [103]. Reliability and validity of the structured questionnaires were examined using Cronbach’s α coefficient and the results (α = 0.946) demonstrated that the questionnaire exhibited high reliability and validity. The quantitative questionnaire surveys were conducted at four points in time including baseline of initial ART, and after 6, 12, and 24 months of ART.

### Statistical analysis

Before data analysis, different surveys and variables were checked for logic using SAS, covering ranges, consistency, logical relationships, and other factors. Whenever such checks revealed errors, original hard copy questionnaires and medical records were revisited for final confirmation and correction. Some participants gave answers of “I don’t know” or made no response at all. These were treated as “no”. All data from different sites were merged into a unified dataset based on the variables in the questionnaire using SAS 9.1.

Sample size calculations were based on the following assumptions: Significance (alpha) was set to 5%; power (beta) to 90%; desired changes in QOL over time to 0.8 for all QOL domains; 20% was allowed for loss to follow up. Given these considerations and calculations, it was determined that a sample size of approximately 300 people infected with HIV was adequate. About 40% of the cohort participants were from Guangxi CDC ART clinic in Nanning City, 30% from the 4th Nanning Hospital ART Clinic, 20% from Pingxiang People’s Hospital ART Clinic, and about 10% from Guigang City People’s Hospital and Guilin 3rd People’s Hospital.

Descriptive analysis was used to describe the demographic characteristics (e.g., age, sex, educational level, marital status, occupation, living status, and monthly household income) of participants. Generalized estimating equations (GEE) with standardized counselling sessions were used to analyse the effect of family support and discrimination on patients’ overall quality of life.

## Results

A total of 332 subjects were recruited for the study. 267 (80.4%), 260 (78.3%), and 246 (74.0%) continued to participate at the 6-month, 12-month, and 24-month periods, respectively (Table 1). Continued participation represented acceptable retention rates. Attrition was attributed to an inability to conduct follow-up, death, and transfer from the study facilities. Of the baseline participants, 226 (68.1%) were male and 106 (31.9%) were female. The average age was 39.6 years (range 20.2–76.5); 42.2 (a range of 19.9–79.5) for men and 38.3 (range 22.2–74.1) for women. Here, 110 participants had a primary school education or less, accounting for 33.2% of the cohort, 150 participants (45.3%) had a secondary school education, and 71 (21.5%) had a high school education or above; 218 (65.7%) were married or cohabitating, and 114 (34.3%) were unmarried and not cohabitating. The majority of participants (62.0%) were farmers or unemployed and 125 (38.0%) were employed. 43 (14.8%) participants currently lived alone. A majority (59.4%) of the patients’ average monthly household incomes were below 1000 RMB (1000 RMB = 144.7 US$). There were no significant changes in the study participants’ demographics over time (Table 2).

**Table 1:** Recruitment information regarding loss to follow up at assessment times

Following up results	6 month		12 month		24 month	
	*N*	%	*N*	%	*N*	%
Completed survey	267	80.42	260	78.31	246	74.10
Died	20	6.02	22	6.63	28	8.43
Transferred out of facility	13	3.92	25	7.53	27	8.13
Lost to follow up	7	2.11	20	6.02	19	5.72
Did not complete survey	25	7.53	5	1.51	12	3.62

**Table 2:** Demographic characteristics of people infected with HIV over time

Items		Baseline		6-month		12- month		24 -month	
		*n*	%	*n*	%	*n*	%	*n*	%
Gender	Male	226	68.07	177	53.31	179	53.92	168	50.60
	Female	106	31.93	90	27.11	81	24.40	78	23.49
	Missing	0	0.00	65	19.58	72	21.69	86	25.90
Age group	20–29	95	28.61	84	25.30	78	23.49	72	21.69
	30–39	105	31.63	86	25.90	88	26.51	79	23.80
	≥ 40	131	39.46	97	29.22	94	28.31	95	28.61
	Missing	1	0.30	65	19.58	72	21.69	86	25.90
Level of education	Primary school or below	110	33.13	77	23.19	76	22.89	76	22.89
	Middle school	150	45.18	125	37.65	123	37.05	110	33.13
	High school or above	71	21.39	64	19.28	61	18.37	60	18.07
	Missing	1	0.30	66	19.88	72	21.69	86	25.90
Marriage status	Married or cohabitating	218	65.66	181	54.52	174	52.41	162	48.80
	Single/divorced/widowed	114	34.34	86	25.90	86	25.90	84	25.30
	Missing	0	0.00	65	19.58	72	21.69	86	25.90
Occupation	Farmer/unemployed township citizens	204	61.45	158	47.59	152	45.78	145	43.67
	Employed	125	37.65	106	31.93	105	31.63	98	29.52
	Missing	3	0.90	68	20.48	75	22.59	89	26.81
Live alone	Yes	43	12.95	32	9.64	31	9.34	30	9.04
	No	247	74.40	205	61.75	200	60.24	184	55.42
	Missing	42	12.65	95	28.61	101	30.42	118	35.54
Monthly household incomes	< 500 RMB	95	28.61	75	22.59	74	22.29	71	21.39
	500 – RMB	88	26.51	69	20.78	68	20.48	65	19.58
	1000 – RMB	125	37.65	101	30.42	100	30.12	97	29.22
	Missing	112	33.73	87	26.20	90	27.11	99	29.82

### Quality of life of ART patients at baseline upon initiation of ART and after 6, 12, and 24 months of ART

At the baseline assessment, average scores on QOL were approximately at middle level: Overall perception of QOL from patients themselves 11.3, overall perception of health from patients themselves 10.3, physical health 12.5, psychological health 11.3, level of independence 11.6, social relationships 11.4, environment 11.4, spirituality 12.0, overall QOL score 11.5.

At the 6-month survey, QOL scores were as follows: overall perception of quality of life 12.6, overall perception of health 12.9, physical health 14.4, psychological health 13.1, level of independence 13.6, social relationships 12.0, environment 12.4, spirituality 13.1, overall QOL score 13.0. All of these average scores were significantly higher after 6 months of ART.

The 12-month survey yielded the following participant perception of QOL: overall perception on QOL 12.6, overall perception on health 13.0, physical health 14.2, psychological health 12.8, level of independence 13.7, social relationships 11.6, environment 12.0, spirituality 12.9, overall QOL score 12.8.

At the 24-month survey, QOL scores were as follows: overall perception on quality of life 13.4, overall perception on health 13.7, physical health 14.8, psychological health 13.6, level of independence 13.8, social relationships 11.8, environment 12.2, spirituality 13.8, and overall QOL score 13.4.

**Fig. 2:**
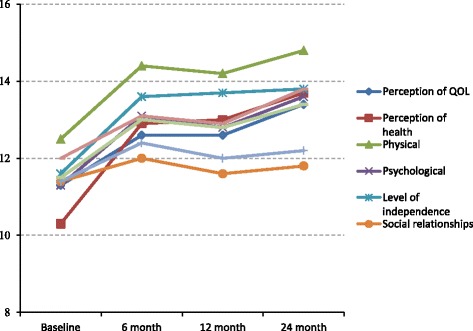
Quality of life score

Some scores, including physical, psychological, social relationship, environment, spiritually, and overall QOL scores were lower at 12 months than at 6 months, although they had increased again by the 24-month survey. All scores were significantly higher at the 24-month follow-up assessment than that at 12-month assessment (Fig. 2).

### Family support and discrimination

Most of the patients (81.3%) disclosed their HIV positive status to family before receiving ART and this proportion increased to 96.7% at the 24-month mark. Among those who had disclosed their HIV positive status to their family, most of them were satisfied with their decision to disclose; 90.4% of patients had received family support at baseline, 91.8% at 6 months, 95.5% at 12 months, and 94.3% at 24 months-significantly higher than that at baseline. Most patients (55.2% and 84.5%) had received encouragement as well as psychological, financial and physical care support from their families at the baseline and at the 24-month survey.

Participants’ perception of family support increased over time. The proportion of patients who did not feel discriminated against by their families was 87.2% at baseline, 90.4% at 6 months, 90.0% at 12 months, and 94.5% at 24 months. At baseline, 28.1% of the patients reported not visiting family because they thought they would not be welcomed, and the rate significantly decreased to 13.3% at the time of the 24 month survey. The rate of patient fear of abandonment by family significantly decreased from 20.7% at baseline to 8.4% at 24 months (Table 3).

**Table 3:** Family support and discrimination over time

Items		Baseline		6 month		12 month		24 month	
		*n*	%	*n*	%	*n*	%	*n*	%
Disclosed HIV positive status to family	Yes	265	81.3	232	92.8	242	93.1	237	96.7
	No or no any family	61	18.7	18	7.2	18	6.9	8	3.3
Family provides supports	No	27	9.6	20	8.2	10	4.5	13	5.7
	Yes	254	90.4	224	91.8	210	95.5	217	94.3
Family provides psychological support	Yes	228	80.3	193	79.1	186	84.5	180	78.3
	No	56	19.7	51	20.9	34	15.5	50	21.7
Family provides financial support	Yes	207	72.9	176	72.1	147	66.8	127	55.2
	no	77	27.1	68	27.9	73	33.2	103	44.8
Family provides physical care support	yes	182	64.1	175	71.7	164	74.5	149	64.8
	No	102	35.9	69	28.3	56	25.5	81	35.2
Satisfaction with decision to disclose HIV+ status to family	Satisfied/Very satisfied	224	84.8	186	80.5	180	86.1	199	90.5
	Somewhat (or not) satisfied	40	15.2	45	19.5	29	13.9	21	9.5
Felt discrimination from family	Yes	35	12.8	22	9.6	21	10.0	12	5.5
	Never	239	87.2	207	90.4	189	90.0	207	94.5
Patients have found themselves not visiting family because patients thought they would not be welcomed	Yes	79	28.1	45	18.7	39	18.1	30	13.3
	Never	202	71.9	196	81.3	176	81.9	195	86.7
Patient fears abandonment by family	Yes	50	20.7	37	16.2	24	12.0	18	8.4
	No	192	79.3	191	83.8	176	88.0	197	91.6

### Effect of family support and discrimination on QOL

GEE analysis was used to study the factors regarding family support and discrimination and their influence on overall quality of life further. Eight factors related to family support and discrimination were significantly associated with the overall quality of life scores: disclosure of HIV positive status, family support, psychological support from family, physical care support from family, satisfaction with decision to disclose HIV positive status, no experience of discrimination by family, continuation of visits to family, no fear of abandonment by family. These all were significantly associated with higher patient QOL scores (Table 4).

**Table 4:** Impact of family support, discrimination, clinical indicators, and demographic characteristics on overall quality of life

Items		*OR*	95% *CI*	*P*
Disclosed HIV positive status to family	No	1.0		
	Yes	1.343	1.02–1.75	0.045*
Family provides support	No	1.0		
	Yes	2.74	1.68–4.47	0.040*
Family provides psychological support	No	1.0		
	Yes	1.356	0.63–1.19	0.040*
Family provides physical care support	No	1.0		
	Yes	2.72	1.41–5.27	0.029*
Satisfaction with decision to disclose HIV+ status to family	Somewhat (or not) satisfied	1.0		
	Satisfied/Very satisfied	1.61	1.18–2.18	0.037*
Felt discrimination from family	Yes	1.0		
	Never	1.30	1.07–1.59	0.041*
Patients have found themselves not visiting family because patient thought they would not be welcomed	Yes	1.0		
	Never	1.65	0.23–1.74	0.035*
Patient fears abandonment by family	Yes	1.0		
	Never	2.05	1.49–2.82	0.025*
Gender	Female	1.0		
	Male	0.72	0.61–0.85	0.056
Education level	No formal education	1.0		
	Primary school	1.05	0.62–1.74	0.932
	Secondary school	0.95	0.57–1.58	0.921
	High school	0.89	0.53–1.50	0.822
	University or above	0.97	.055–1.72	0.963
Marital status	Married and cohabitating	1.0		
	Cohabiting but not married	0.73	0.53–1.00	0.321
	Never married and not cohabitating	1.60	1.18–2.18	0.122
	Divorced/separated & not cohabitating	0.81	0.62–1.05	0.409
	Widowed and not cohabitating	1.91	1.10–3.31	0.242
Income	–	1.63	1.04–2.57	0.281
Age	–	1.00	0.99–1.01	0.978
CD4 cell count	–	1.22	0.87–1.71	0.559
AIDS staging	1	1.0		
	2	0.89	0.59–1.36	0.786
	3	0.73	1.31–1.56	0.321
	4	0.81	0.66–1.14	0.317

* means *P* < 0.05 and there were statistically significance

## Discussion

In the study, results showed the percentage of participants who reported that they felt discrimination from family to be smaller than in other countries [32, 33], and it decreased significantly from 12.8% at baseline to 5.5% at 24 months. Moreover, the quality of life scores increased alongside increases in family support and decreased in response to discrimination from family during ART treatment. However, the speed and magnitude of improvement were greatest during the first 6 months after ART initiation. They plateaued between 6 and 12 months for most QOL domains. Improved physical symptoms based on CD4 cell counts, hemoglobin, total lymphocyte counts, creatinine (UI/L), amylase, and lipase at the baseline may explain the initial decrease and subsequent increase in domains, such as physical health, psychological health, social relationships, and spirituality. Moreover, results showed that the demographics and experiences of the initial participants who left the study were representative of the entire population studied. That is, the attrition rate did not affect the results. Many HIV-infected patients (75.7%) had already developed symptoms when they began ART; Zhang reported that approximately 81% of HIV-infected patients in China had symptoms when they began ART [30]. Another study indicated that the QOL of patients with symptoms would see more pronounced improvements than those without symptoms after ART [33]. Generally, with the increase of therapy time, the QOL of people infected with HIV showed a rising trend from baseline to 24 months. However, more in-depth research is needed to better understand the underlying causes for the plateau and, in some cases, decrease. In particular, greater attention should be paid to patients after 6 months in order to continuously improve patient quality of life. A positive diagnosis of HIV can also increase the discrimination from the patients themselves, which entails not only physical but also significant psychosocial consequences. In the study, 20.7% of people infected with HIV feared abandonment by their families, 28.1% of patients at baseline have found themselves not visiting family because they thought they would not be welcome, and there were significant differences in discrimination from patients themselves and QOL. HIV’s involvement in the central nervous system and other parts of the physical body are particularly stressful for patients themselves because the infection is incurable and usually accompanied both by social stigma and symptoms that impair both cognitive and physical function. These fears may become significant enough to stress social and family relationships, which adversely affects the quality of care, treatment outcomes, and QOL of people infected with HIV.

GEE analysis revealed that nearly all of the family factors were significantly closely associated with overall quality of life scores of the patients. Among these factors, whether the patient has disclosed his or her HIV status to family, whether the family provided supports, and whether the patients were satisfied or very satisfied with their decision to disclose their HIV-positive status to family were associated with higher overall quality of life scores. These results remind us that in order to improve overall patient quality of life, especially in the context of Chinese culture, we must actively provide counselling services to encourage patients to disclose their HIV status to their families earlier. Because families can only provide the support that their HIV-positive relatives need if they know that it is needed, disclosure to family members is imperative, and strategies to encourage people infected with HIV to disclose their health conditions to family after diagnosis must be developed. For example, the community, especially clinicians who work with families affected by HIV, need to address the discrimination and stigma issues associated with disclosure in order to increase social support in an attempt to reduce any negative emotional impact that disclosure may have on people infected with HIV. In addition, many studies have suggested that, in general, there is a positive relationship between income and quality of life [34, 35]. In this study, results showed there is no statistically significant relationship between family income and quality of life (*P* > 0.05) although family income was found to vary slightly across different periods.

In this study, most participants disclosed their HIV status to family members and the rates increased significantly from 81.3% at baseline to 96.7% at 24 months. More than 90% of the participants received support from their families and the proportion remained high at 12-months and 24-months. These results indicated that, as has been found in Chinese culture, family were the people the patients were most likely to trust and to play the most important role in providing support [36]. Therefore, more efforts must be made to reduce the discrimination and stigma associated with HIV-positive status from patients’ families. Additionally, patients’ own doubts regarding their sense of belonging negatively influenced their overall quality of life. For example, many patients who did not limit family visits because they believed that they would not be welcomed or did not fear abandonment by family enjoyed a greater overall quality of life. This suggests that more psychological support and counselling to reduce patients’ self-perceived stigma and discrimination is also important. Moreover, greater effort should be made to provide training and support to the families of PLHIV so that patients receive more effective home-based care and support. These efforts could include mobilizing volunteers to visit families of individuals with AIDS, educating co-workers and family members about basic care issues and putting them in touch with referral networks of health facilities and welfare agencies, assisting with household chores, and providing psychological support.

### Limitations

The present study has some limitations that should be acknowledged. The respondents were recruited solely among individuals actively seeking routine medical care. Those who did not keep regular clinic appointments or peer organization visits could not be included. The results of this study may not be generalizable to all HIV-positive people in China. Moreover, the face-to-face interviews, the assessment of readiness with regard to family and peer support and the administration of the questionnaire by the research team may have led to social desirability biases or affected QOL scores and related results. Nurses, counsellors, and research staff were trained to conduct the interviews in a non-judgmental and objective way and the changes over time were consistent across study sites. Missed observations due to participants dropping out of the study or missed appointments could have caused outcome bias. However, a weighted GEE method was used based on the bias parameters regarding missing data, and the cases of demographics and experiences of the initial participants who left the study were analysed. Overall the results indicated that the participants who left the study could be representative of the entire population studied. In this way, the attrition rate may not have affected the results. Self-reporting data may also have introduced bias. We did not identify any specific association between young men and the fact that about 15% of all people infected with HIV are young people aged 15 to 24. Further research focusing on family support and young people is urgently needed.

## Conclusions

Family support and either no or very limited discrimination was found to contribute to QOL among people infected with HIV, which tended to improve significantly as ART continued. Strategies to improve and strengthen family support and care for PLWHA and promote HIV screening among high-risk populations should be developed and implemented by both policy makers and researchers. For example, the community, especially clinicians who work with families affected by HIV, must encourage high-risk individuals to undergo HIV testing regularly and to address discrimination and stigma associated with disclosure in order to encourage early initiation of ART and increase the QOL of PLWHA.

## Additional file

Additional file 1: Multilingual abstracts in the five official working languages of the United Nations. (PDF 735 kb)

## Abbreviations

ART: antiretroviral therapy
GEE: generalized estimating eqs.
HR-QOL: health-related quality of life
PLWHA: people living with HIV/AIDS
QOL: quality of life
