# Supplementary material for: Family support, discrimination, and quality of life among ART-treated HIV-infected patients: a two-year study in China
Source: Infect Dis Poverty. 2017 Nov 21;6:152. doi: 10.1186/s40249-017-0364-5 (PMC5697335; doi:10.1186/s40249-017-0364-5)
Supplement: Additional file 1: — Multilingual abstracts in the five official working languages of the United Nations. (PDF 735 kb) [file 40249_2017_364_MOESM1_ESM.pdf]

Translation of the abstract into the five official working languages of the United Nations

الدعم الأسري، والتمييز، ونوعية الحياة بين المصابين بفيروس نقص المناعة البشرية والخاضعين للعلاج بمضادات فيروسات النسخ العكسي : وذلك بحسب دراسة صينية منذ عامان.

شون فانغ زو، وتشيانغ تشونغ مينغ، وتشانغ تشيان زانج، وبي تشنغ وانغ، وجون جينغ وفنغ تشنغ

ملخص

لمحة عامة بحلول أيلول/سبتمبر 2016، كان يعيش زهاء 865 653 مصاب بفيروس نقص المناعة البشرية/الإيدز في الصين،: في حين أن كان هناك 725 492 مريض يتلقون علاجًا بمضادات الفيروسات الرجعية (أرت). كثيرًا ما يعاني هؤلاء المصابون بمرض نقص المناعة البشرية \ الإيدز من التفرقة والتمييز في كافة المجالات في حياتهم الشخصية والاجتماعية. وقد أدرجت منظمة الصحة العالمية تلك التفرقة التي يتلقونها ضمن قائمة العوامل الاجتماعية خاصتها الخاصة بعوامل الصحة المرتبطة بالصحة النفسية والعقلية المريضة وغير السليمة. كما يضم هذا البحث بين طياته الدعم الأسري والتفرقة التي يتعرض لها هؤلاء المرضى المصابين بفيروس نقص المناعة البشرية، ويدرس تأثير كل منها على حياة المرضى (QOL) ومدى تأثرها بذلك من خلال خضوعهم للعلاج بمضاد الفيروسات الرجعية في الصين.

الأساليب: وقد أجرينا هذه الدراسة الجماعية الرقابية على علاج مضاد الفيروسات الرجعية- المرضى المصابين بفيروس نقص المناعة البشرية الذين تم علاجهم في مقاطعة قوانغشي باستخدام مسح استبيان عند خط اساس، 6 و 12 و 24 شهرًا، اعتبارًا من عام 2010. كما استُخدِمَ التحليل الوصفي لوصف الخصائص الديموغرافية (مثل السن والنوع والمستوى التعليمي والحالة الاجتماعية والوظيفية) للمشاركين بالدراسة. وقد استُخدمت المعادلات التقديرية المعممة (GEE) لدراسة العلاقات بين دعم الأسرة، والتمييز، ونوعية الحياة.

النتائج: أظهرت الدراسة أن 90.4 % من المرضى (*حجم العينة*=281 مريضًا) قد تلقوا الدعم الأسري في الوضع الحالي، المُعرف هنا بأنه الشروع في العلاج بمضادات الفيروسات الرجعية، في حين تلقى 91.8% من المرضى (*حجم العينة*=244مريضًا) الدعم الأسري بعد الشروع في العلاج بمضادات الفيروسات الرجعية بستة أشهر، كما تلقى 95.5% من المرضى (*حجم العينة*= 220 مريضًا) الدعم الأسري بعد الشروع في العلاج بمضادات الفيروسات الرجعية باثنتي عشر شهرًا، وأخيرًا تلقى 94.3 % (*حجم العينة*=230 مريضًا) الدعم بعدها بأربعة وعشرين شهرًا. النتائج:<bx id="2"/></g>أظهرت الدراسة أن 90.4 % من المرضى (<g id="3">حجم العينة</g>=281 مريضًا) قد تلقوا الدعم الأسري في الوضع الحالي، المُعرف هنا بأنه الشروع في العلاج بمضادات الفيروسات الرجعية، في حين تلقى 91.8% من المرضى (<g id="4">حجم العينة</g>=244مريضًا) الدعم الأسري بعد الشروع في العلاج بمضادات الفيروسات الرجعية بستة أشهر، كما تلقى 95.5% من المرضى (<g id="5">حجم العينة</g>= 220 مريضًا) الدعم الأسري بعد الشروع في العلاج بمضادات الفيروسات الرجعية باثنتي عشر شهرًا، وأخيرًا تلقى 94.3 % (<g id="6">حجم العينة</g>=230 مريضًا) الدعم بعدها بأربعة وعشرين شهرًا. وقد ارتبطت النتائج الكلية نوعية الحياة التي أجريت على عشرات المرضى بشكل إيجابي مع دعم الأسرة (*أو* = 2.74، ف = 0.040، 95% *CI* = 1.68 – 4.47)، الشعور بعدم وجود تفرقة أو تمييز داخل الأسرة(*OR* = 1.3، ف = 0.041، 95% *CI* = 1.07 – 1.59) أو التمييز من المرضى أنفسهم، بما في ذلك عدم المعاناة من الخوف من هجر الأسرة(*أو* = 2.05، ف = 0.025، 95% *CI*: 1.49 – 2.82).

الاستنتاجات: <bx/>وُجد أن الدعم الأسري جنبًا إلى جنب مع عدم التمييز اوالحد الأدنى من التمييز من شأنه ان يبث الحياة في نفوس المرضى المصابين بفيروس نقص المناعة البشرية. فضلاً عن أن نوعية حياتهم بشكل عام كانت تميل إلى التحسن كثيرًا مع مواصلة العلاج الأمر الذي يشير إلى ضرورة أن يعمل كلّ من صناع السياسات والباحثين على دراسة الاستراتيجيات التي ترمي إلى تحسين الدعم الأسري وتعزيزه، ورعاية الأشخاص الذين يتعايشون مع فيروس نقص المناعة البشرية (PLWHA)، وتحسين فحوصات فيروس نقص المناعة البشرية لدى السكان الأكثر عرضة للإصابة به.

Translated from English version into Arabic through

艾滋病抗病毒治疗患者的家庭支持、歧视和生活质量：一项来自中国的两年纵向研究

徐俊芳，明中强，张昱乾，王培成，景军，程峰

摘要
引言：截止 2016 年 9 月，全国报告现存活艾滋病病毒感染者/AIDS 病人 653 865 例，累计 492 725 例正在接受抗病毒治疗。艾滋病病毒感染者/AIDS 病人在生活中经历着来自各方面的歧视和侮辱。世界卫生组织已经把歧视列为影响生理和心理健康的社会决定因素。随着抗病毒治疗的不断进展，本研究探讨了艾滋病感染者的家庭支持和歧视情况，并分析了家庭支持和歧视对其生活质量的影响。
方法：采用观察性的队列研究方法，选择广西作为研究现场，运用问卷调查的方法从 2010 年开始对接受抗病毒治疗的艾滋病病毒感染者进行了 3 次问卷调查（包括治疗前的基线问卷调查、开始治疗后的第 6 个月、第 12 个月和 24 个月的随访问卷调查）。并采用描述性分析描述参与者的人口学特征（如年龄、性别、教育程度、婚姻状况和职业状况）。同时采用广义估计方程探讨家庭支持、歧视与生活质量之间的关系。
结果：在本研究中，90.4%（n = 281）的艾滋病病毒感染者在基线时（即开始抗病毒治疗时）获得了家庭支持，抗病毒治疗 6 个月以后，91.8%（n = 244）的取得了家庭支持，12 个月以后达到 95.5%（n = 220），24 个月后为 94.3%（n = 230）。在基线时，未感受到家庭歧视的艾滋病病毒感染者的比例为 87.2%（n = 274），抗病毒治疗 6 个月后为 90.4%（n = 229），12 月后为 90%（n = 210），24 个月后达到 94.5%（n = 219）。艾滋病患者的总体生活质量得分与获得的家庭支持（OR = 2.74，P = 0.040，95%CI: 1.68 - 4.47），没有感受到来自家庭的歧视（OR = 1.3，P = 0.041，95%CI: 1.07 - 1.59），以及感染者没有自身歧视(包括从未害怕过家庭的抛弃)（OR = 2.05，P = 0.025，95%CI: 1.49 - 2.82）呈显著正相关。
结论：家庭支持以及尽量减少家庭的歧视有利于提高*艾*滋病病毒感染者的生活*质量*。随着抗病毒治疗的继续，艾滋病患者的总体生活质量有了显著的提高。研究表明决策者和研究者应探索改善和加强艾滋病病毒感染者的家庭支持以及促进高危人群艾滋病筛查的策略。

Translated from English version into Chinese by Jun-Fang Xu

Soutien familial, discrimination et qualité de vie parmi les patients infectés par le VIH sous antirétroviraux : une étude de deux ans en Chine

Xu Jun-Fang, Ming Zhong-Qiang, Zhang Yu-Qian, Pei-Cheng Wang, Jun Jing et Feng Cheng

Résumé
Contexte: À la date de septembre 2016, environ 653 865 personnes étaient séropositives au VIH ou atteintes du SIDA en Chine et 492 725 suivaient un traitement antirétroviral (TARV). Les patients séropositifs sont souvent victimes de discriminations dans tous les domaines de leur vie personnelle

et sociale. L'Organisation mondiale de la Santé inclut la discrimination dans sa liste de déterminants sociaux des facteurs de santé liés à une mauvaise santé physique ou psychologique. Notre article examine le soutien familial dont bénéficient les séropositifs chinois et les discriminations qu'affrontent ceux-ci, ainsi que leur effet sur la qualité de vie des patients sous traitement antirétroviral.

Méthodes: Nous avons mené cette étude observationnelle de cohorte sur des patients séropositifs au VIH sous TARV dans la province du Guangxi, à l'aide d'un questionnaire présenté au début de l'étude et à 6, 12 et 24 mois, à partir de 2010. Nous avons procédé à une analyse descriptive afin de décrire les caractéristiques démographiques des participants (par ex. âge, sexe, niveau d'éducation, situation de famille, situation professionnelle). Des équations d'estimation généralisées (GEE) ont été utilisées pour examiner les relations entre soutien familial, discriminations et qualité de vie.

Résultats: Dans cette étude, 90,4 % (*n* = 281) des patients bénéficiaient du soutien de leur famille au début de l'étude (défini comme la mise en place du TARV), 91,8 % (*n* = 244) 6 mois après le début du TARV, 95,5 % (*n* = 220) à 12 mois et 94,3 % (*n* = 230) à 24 mois. La proportion de patients qui ne pensaient pas souffrir de discrimination de la part de leur famille était de 87,2 % (*n* = 274) au début de l'étude, 90,4 % (*n* = 229) 6 mois après le début du traitement, 90,0 % (*n* = 210) à 12 mois et 94,5 % (*n* = 219) à 24 mois. Les scores de qualité de vie globale des patients étaient associés positivement au soutien familial(*OR* = 2,74, *P* = 0,040, IC à 95 % = 1,68 à 4,47), au fait de ne pas ressentir de discrimination de la part de la famille(*OR* = 1,3, *P* = 0,041, IC à 95 % = 1,07 à 1,59) ni de la part des patients eux-mêmes, et notamment au fait de n'avoir jamais craint d'être abandonné par sa famille (*OR* = 2,05, *P* = 0,025, IC à 95 % = 1,49 à 2,82).

Conclusions: Le soutien familial et l'absence de discrimination (ou une discrimination minimale) s'avèrent contribuer à la qualité de vie des patients infectés par le VIH, qui tend à s'améliorer significativement au fil du TARV. Ces résultats donnent à penser que les décideurs politiques comme les chercheurs devraient explorer que des stratégies destinées à améliorer et renforcer le soutien familial, prendre soin des séropositifs et encourager le dépistage du VIH dans les populations à haut risque.

Translated from English version into French by Suzanne Assenat, through

TRANSLATORS
WITHOUT BORDERS

Семейная поддержка, дискриминация и качество жизни среди ВИЧ-инфицированных пациентов, прошедших антиретровирусную терапию (АРТ): двухлетнее исследование, проведённое в Китае

Цзюнь-Фан Сюй, Чжун-Цян Мин, Юй-Цянь Чжан, Пэй-Чэн Ван, Цзюнь Цзин и Фэн Чэн

Выдержка

Справочная информация: по состоянию на сентябрь 2016 года в Китае примерно 653 865 человек были заражены ВИЧ/СПИДом (ЛЖВС), и 492 725 человек получали

антиретровирусную терапию (АРТ). ЛЖВС (люди, живущие с ВИЧ и СПИДом) часто сталкиваются с дискриминацией во всех областях своей личной и общественной жизни. Всемирная организация здравоохранения включает дискриминацию в свой перечень социальных определяющих факторов здоровья, влияющих на физическое и психологическое состояние. В данном исследовании рассматривается как семейная поддержка, так и дискриминация, с которой сталкиваются люди, инфицированные ВИЧ, а также проводится анализ воздействия указанных факторов на качество жизни пациентов (КЖП) по мере прохождения ими антиретровирусной терапии в Китае.

Методы: Данное обсервационное когортное исследование ВИЧ-инфицированных пациентов–, получающих антиретровирусную терапию, было проведено в автономном районе Гуанси при помощи анкетирования, проведённого через 6, 12 и 24 месяца, начиная с 2010 года. Для описания демографических характеристик (включая возраст, пол, уровень образования, семейное положение и занятость) участников был задействован описательный анализ. Для изучения взаимосвязи между семейной поддержкой, дискриминацией и КЖП был использован метод обобщенного оценочного уравнения (GEE).

Результаты: В данном исследовании 90,4% (*n* = 281) пациентов получали семейную поддержку с начального момента, указаного здесь как начало проведения АРТ-терапии, тогда как 91,8% (*n* = 244) получали поддержку семьи спустя 6 месяцев после начала проведения АРТ-терапии, 95,5% (*n* = 220) после 12 месяцев и 94,3% (*n* = 230) после 24 месяцев. Доля пациентов, которые не испытывали дискриминации в своих семьях, составила 87,2% (*n* = 274) на начальный момент, 90,4% (*n* = 229) через 6 месяцев после начала проведения АРТ-терапии, 90,0% (*n* = 210) по прошествии 12 месяцев и 94,5% (*n* = 219) по истечении 24 месяцев. Общие показатели КЖП пациентов были положительно связаны с получением поддержки семьи (*OR* = 2,74; *P* = 0,040, 95% *CI* = 1,68-4,47); отсутствием дискриминации со стороны их семей (*OR* = 1,3; *P* = 0,041; 95% *CI* = 1,07 – 1,59) или дискриминации от самих пациентов, включая полное отсутствие страха быть покинутым своей семьёй (*OR* = 2,05; *P* = 0,025; 95% *CI*: 1,49 – 2,82).

Выводы: Поддержка семьи, наряду с полным отсутствием или минимальной дискриминацией, влияет на КЖП людей, инфицированных ВИЧ. Общее КЖП в их случае, как правило, значительно улучшалось по мере проведения антиретровирусной терапии (АРТ). Это свидетельствует о необходимости проведения исследований как со стороны ученых, так и со стороны политических деятелей в области стратегий, направленных на улучшение и укрепление поддержки семьи, уход за ЛЖВС, а также на содействие в проведении проверок на ВИЧ среди населения, входящего в группу повышенного риска.

Translated from English version into Russian through

TRANSLATORS
WITHOUT BORDERS

Apoyo familiar, discriminación y calidad de vida entre los pacientes infectados por el VIH y tratados con TAR: estudio de dos años realizado en China

Autores: Jun-Fang Xu, Zhong-Qiang Ming, Yu-Qian Zhang, Pei-Cheng Wang, Jun Jing y Feng Cheng

Resumen

Contexto: En China, en septiembre de 2016, unas 653 865 personas convivían con el VIH/SIDA, de las cuales 492 725 recibían terapia antirretroviral (TAR). Con frecuencia, los seropositivos sufren discriminación en todos los ámbitos de su vida personal y social. La Organización Mundial de la Salud incluye la discriminación entre los determinantes sociales de la salud que se han relacionado con problemas de salud físicos y psicológicos. Este artículo identifica el apoyo familiar que reciben las personas infectadas por el VIH y la discriminación a la que se enfrentan, y estudia el efecto de ambos en la calidad de vida de los pacientes de China tratados con TAR.

Métodos: Se realizó un estudio observacional de cohorte de TAR-Utilizando como punto de partida un cuestionario, se trató a los pacientes con VIH de la provincia de Guangxi durante seis, doce y 24 meses a partir de 2010. Para describir las características demográficas (edad, sexo, nivel educativo, estado civil y situación laboral) de los participantes se utilizó el análisis descriptivo. Para estudiar la relación entre apoyo familiar, discriminación y calidad de vida se utilizaron ecuaciones de estimación generalizada (EEG).

Resultados: En el estudio, el 90,4 % (*n* = 281) de los pacientes recibieron apoyo familiar al inicio -definido aquí como el comienzo de la terapia-, el 91,8 % (*n* = 244) seguían contando con dicho apoyo a los seis meses de haber empezado, el 95,5 % (*n* = 220), a los 12 meses, y el 94,3 % (*n* = 230), a los 24 meses. La proporción de pacientes que no se sentían discriminados por sus familias era del 87,2 % (*n* = 274) al inicio de la terapia, el 90,4 % (*n* = 229) a los seis meses, el 90,0 % (*n* = 210) a los 12 meses, y el 94,5 % (*n* = 219) a los 24 meses. Las valoraciones generales de la calidad de vida de los pacientes se asociaron positivamente a haber recibido apoyo familiar (*OR* = 2,74, *P* = 0,040, 95 % *IC* = 1,68 - 4,47), a no sentirse discriminados por sus familias (*OR* = 1,3, *P* = 0,041, 95 % *IC* = 1,07 - 1,59) ni por ellos mismos; en particular a no haber experimentado nunca temor a que su familia los abandonase (*OR* = 2,05, *P* = 0,025, 95 % *IC*: 1,49 - 2,82).

Conclusiones:</bx>Se determinó que el apoyo familiar, junto con una discriminación mínima o inexistente, contribuía a la calidad de vida de las personas infectadas por el VIH. La calidad general de vida tendía a mejorar considerablemente a medida que avanzaba la terapia. Esto sugiere que tanto los responsables políticos como los investigadores deberían estudiar estrategias para mejorar y fortalecer el apoyo familiar y la atención a este tipo de pacientes, así como para promover la detección del VIH entre grupos de alto riesgo.

Translated from English version into Spanish through

TRANSLATORS WITHOUT BORDERS
